# Multiple Stressors in a Top Predator Seabird: Potential Ecological Consequences of Environmental Contaminants, Population Health and Breeding Conditions

**DOI:** 10.1371/journal.pone.0131769

**Published:** 2015-07-14

**Authors:** Jan O. Bustnes, Sophie Bourgeon, Eliza H. K. Leat, Ellen Magnusdóttir, Hallvard Strøm, Sveinn A. Hanssen, Aevar Petersen, Kristin Olafsdóttir, Katrine Borgå, Geir W. Gabrielsen, Robert W. Furness

**Affiliations:** 1 Norwegian Institute for Nature Research (NINA), Fram Centre, 9296, Tromsø, Norway; 2 Norwegian Polar Institute, Fram Centre, 9296, Tromsø, Norway; 3 College of Medical, Veterinary and Life Sciences, Graham Kerr Building, University of Glasgow, Glasgow, G12 8QQ, United Kingdom; 4 University of Iceland, Department of Pharmacology & Toxicology, IS-107, Reykjavik, Iceland; 5 Brautarland 2, 108, Reykjavik, Iceland; 6 Department of Biosciences, University of Oslo, P.O. Box, 1066 Blindern, 0316, Oslo, Norway; Institute of Ecology, GERMANY

## Abstract

Environmental contaminants may have impacts on reproduction and survival in wildlife populations suffering from multiple stressors. This study examined whether adverse effects of persistent organic pollutants (POPs) increased with poor population health and breeding conditions in three colonies (60–74°N) of great skua (*Stercorarius skua*) in the north-eastern Atlantic (Shetland, Iceland and Bjørnøya [Bear Island]). POPs (organochlorines [OCs] and polybrominated diphenyl ethers [BDEs]) were measured in plasma of incubating birds (*n* = 222), concentrations differing nearly tenfold among colonies: Bjørnøya (2009) > Bjørnøya (2010) > Iceland (2009) > Shetland (2009). Reproductive success (hatching success and chick survival) showed that breeding conditions were favourable in Shetland and at Bjørnøya (2010), but were very poor in Iceland and at Bjørnøya (2009). Biomarkers indicated that health was poor in the Shetland population compared to the other populations. Females whose chicks hatched late had high POP concentrations in all colonies except at Bjørnøya (2010), and females losing their eggs at Bjørnøya (2009) tended to have higher concentrations than those hatching. Moreover, there was a negative relationship between female POP concentrations and chick body condition at hatching in Iceland and at Bjørnøya (2010). Supplementary feeding experiments were conducted, and in Iceland where feeding conditions were poor, significant negative relationships were found between female POP concentrations and daily growth-rate in first-hatched chicks of control nests, but not in food supplemented nests. This suggests that negative impacts of POPs were mitigated by improved feeding conditions. For second-chicks, there was a strong negative relationship between the female POP concentrations and growth-rate, but no effects of supplementary feeding. Lowered adult return-rate between breeding seasons with increasing POP loads were found both at Bjørnøya (2009) and in Shetland, especially related to BDEs. This indicates stronger fitness consequences of POPs following seasons with very poor breeding conditions and/or high reproductive effort. This study suggests that the impacts of POPs may differ depending on population health and breeding conditions, and that even low concentrations of POPs could have ecological consequences during adverse circumstances. This is important with regard to risk assessment of biomagnifying contaminants in marine ecosystems.

## Introduction

Environmental pollutants may affect both reproduction and survival in wildlife, but their impact on different life-history traits may vary. For example, in long-lived species effects of pollutants are expected to be least severe on life-history traits most important for population growth rate, such as adult survival [1]. Moreover, wild animals face stress of both natural and anthropogenic origin, and multiple stressors may be more detrimental than single stressors acting alone [2], [3]. This implies that even low concentrations of toxic chemicals can be harmful in populations suffering high natural stress (e.g. starvation) and /or in populations dominated by weakened individuals. Hence, to assess the ecological impact of toxicants in a population may be very difficult without simultaneously assessing its health and the current conditions under which it lives [4], [5].

The marine environment is a sink for persistent organic pollutants (POPs) such as organochlorines (OCs), many of which were banned in most countries several decades ago (e.g. PCB and DDT), and emerging compounds, which include brominated flame retardants. Many studies have found associations between POPs and biochemical or physiological traits in seabirds [6], but few studies have documented effects on reproduction and survival. In the glaucous gull (*Larus hyperboreus*) in the Norwegian Arctic, however, high concentrations of legacy POPs were associated with behavioural impairment, poor reproductive performance and lowered survival [7], [8]. Interactions between POPs and natural stressors were also found as POPs were negatively associated with chick growth in females with high feeding costs (flying distance), but not in females with low costs [9]. Moreover, experiments suggested that high parasite loads also triggered reproductive effects of POPs [10]. In great black-backed gulls (*Larus marinus*), adverse relationships between fitness traits and POPs were found when breeding conditions were poor, whereas birds seemed unaffected during good conditions [5]. Nevertheless, there is a paucity of experimental studies in seabirds examining the interactive relationships between breeding conditions and POPs.

The objective of this study was to examine, both observationally and experimentally, how ecological impacts of environmental contaminants in seabirds may vary, predicting worse effects during adverse conditions and when population health is poor. The great skua (*Stercorarius skua*), a large omnivorous seabird with high POP loads [11], [12], was studied in three colonies (Shetland, Iceland and Bjørnøya [Bear Island] in the Norwegian Arctic) over a temperate-arctic gradient in the north-eastern Atlantic (60–74°N). The study population at Foula in Shetland declined by ~30% between 2000 and 2007 due to sustained food shortage and poor breeding success [13], [14], [15]. In contrast, the population at Bjørnøya has grown continuously since its establishment in the 1970s (H. Strøm, unpublished data). Biomarkers of health were recorded in these colonies, showing that birds in the Shetland population were more stressed and had poor immune function compared to birds in the other two colonies in 2009 (Table 1) [16].

**Table 1:** Schematic trends in multiple stressors in breeding great skuas: anthropogenic (POPs) and natural stressors (population health, breeding conditions), including fitness variables.

Multiple stressors			Shetland	Iceland	Bjørnøya	
			2009	2009	2009	2010
	Anthropogenic stressors:	POPs	+	++	++++	+++
	Natural stressors:	Population health	-	++	+++	
		Breeding condition during incubation	++	++	—	++
		Breeding condition during chick rearing	++	—	—	++
	Fitness	Fledging success	++	—	—	++
		Return-rate	86%	71%	80%	90%

POPs (OCs and brominated flame retardants; *i*.*e*. polybrominated diphenyl ethers [PBDEs]) were measured in blood plasma of incubating skuas, and potential reproductive effects of contaminants were analysed in females; firstly the relationships between POP concentrations and 1) hatching-date, and 2) the chick body condition at hatching, since these traits are likely to be affected by toxic chemicals [5], [17], [18], [19]. Secondly, experimental nests were subject to supplementary feeding in all colonies, and the growth rate of the chicks was compared to control nests. We predicted that if nutritional stress influenced the adverse effects of POPs, the impact would be lower in the experimental nests. Finally, the relationship between annual return-rate and POPs was analysed for both sexes to document long-term effects.

## Materials and Methods

The study was carried out at Bjørnøya, Svalbard (74°21’N, 19°05’E), at Öræfi, Iceland (63° 57 ‘N, 16°24’ W) and at Foula, Shetland (60°08 ‘N, 2°05’ W). The numbers of breeding pairs of great skua in the colonies were approximately 350, 1500 and 1660, in Bjørnøya, Öræfi and Foula, respectively [15], [20] [21]. In 2009, a total of 161 incubating adult birds were caught on their nests using remote controlled noose traps, whereas in 2010, only Bjørnøya was studied (*n* = 61; Table 2). Birds were ringed with both metal and colour plastic rings with individual codes. Body mass (± 5 g) and head and bill lengths (± 1 mm) were measured and used in the analyses. At each capture, birds were blood sampled from the brachial or tarsal vein using heparinised syringes (under appropriate national licences in each location). Blood was immediately transferred into Cornic tubes that were stored on ice, centrifuged within 2 h (5000 rpm), with plasma and RBCs frozen and stored at -20°C. Birds were sexed from red blood cells (RBCs) or feather pulp after DNA extraction and PCR amplification of CHD genes using primers 2550F [22] and 2757R (R. Griffiths pers. comm.).

**Table 2:** Summary data for great skuas breeding in different north-eastern Atlantic colonies.

							Bjørnøya						
	Shetland (2009)			Iceland (2009)			2009			2010			P-value
Trait													
	n	mean	Std.Err	n	mean	Std.Err	n	mean	Std.Err	n	mean	Std.Err	
**FEMALES**													
∑OC	35	254.5	29.2	42	621.7	83.3	41	2343.6	217.0	43	1016.6	109.8	<0.0001
∑BDE	35	5.2	0.6	42	8.6	1.1	41	21.8	5.0	43	4.3	0.4	<0.0001
Blood lipid (%)	35	0.86	0.03	42	0.84	0.03	41	0.94	0.02	43	0.76	0.03	<0.0001
Hatching date (date)*	35	27.7	1.1	39	23.5	0.7	33	16.8	1.2	39	4.3	1.1	<0.0001
Clutch size	35	1.94	0.04	41	2	0	41	1.51	0.08	43	2	0	<0.0001
Volume egg A (ml)	34	79.6	0.9	41	84.1	0.9	41	81.1	0.9	43	88.1	1.1	0.0003
Volume egg B (ml)	33	75.9	1.1	41	83.6	0.9	21	78.3	1.5	43	85.6	1.2	0.0005
Hatching success (%)	35	88.6		42	92.9		41	39.0		43	90.7		
Hatching weight chick A (g)	30	61.0	0.8	38	64.9	0.8	18	62.4	1.1	39	67.4	1.3	0.0042
Hatching weight chick B (g)	17	58.8	1.2	32	63.6	0.8	5	61.6	3.2	30	67.2	1.5	0.16
Adult body mass (g)	31	1413.2	12.7	42	1459.9	14.6	41	1443.5	12.0	42	1454.2	18.1	0.63
**MALES**													
∑OC	16	419.9	85.4	17	654.2	99.3	10	2872.4	586.6	18	1272.7	217.4	0.0011
∑BDE	16	7.0	0.8	17	11.2	1.5	10	41.6	17.6	18	6.8	1.0	0.0002
Blood lipid (%)	16	0.91	0.05	17	1.02	0.02	10	0.85	0.07	18	0.85	0.05	0.42
Adult body mass (g)	14	1262.9	18.6	17	1277.0	17.8	10	1278.0	15.8	18	1339.7	23.0	0.07
**Adult return rate (%)**													
Females	35	0.91		41	0.71		41	0.85		43	0.88		
Males	16	0.75		15	0.73		10	0.60		18	0.94		
Total	51	0.86		56	0.71		51	0.80		61	0.90		

* = Average date in June for Iceland and Shetland and July for Bjørnøya.

Return rate between years for Shetland and Iceland was established by checking the colonies repeatedly the following year (i.e., 2010). At Bjørnøya, an on-going monitoring study enabled us to check for returning birds in 2010, 2011 and 2012.

Two days prior to hatching, pairs were randomly selected and given 100 g of beef meat (cat food; Landlord, Reitangruppen, Germany) daily (experimental birds), whereas control pairs were not provided extra food, but were visited at the same frequency to exert the same stress. Food was distributed during day time from the moment when one egg was starting to crack [23].

To make sure that all chicks were found, the surroundings of the nests were thoroughly searched. Chicks were weighed (± 1 g) and body measures (tarsus and wing lengths, ± 1 mm) were recorded every 5 days from hatching up to day 20. We kept track of the hatching rank, i.e., the ranking order with the first hatched chick of the brood being referred to as the A-chick, while the last hatched chick was referred to as the B-chick.

In 2009, the breeding situation at Bjørnøya was so poor that very few birds were able to hatch their eggs, and the experiment could not be successfully completed. In 2010, the experiment was, however, successfully carried out. To achieve a sufficient sample size to compare chick growth between fed and control chicks, we used daily growth rate (g/d) from hatching until day 10 of age.

All sampling procedures and experimental manipulations were reviewed and approved as part of obtaining field permits. In Shetland, all procedures (i.e., remote controlled noose trapping, ringing, blood sampling and experimental feeding) were carried out under licenses from the Home Office (PPL 60/3835 awarded after ethical review of the protocol by The Home Office inspectorate) and the British Trust for Ornithology. In Bjørnøya, all procedures were carried out under permits from the Governor of Svalbard (2009/00103-17 and 2010/00093-16), and ringing license from Stavanger Museum (licensed to H. Strøm). In Iceland, all procedures were covered by the general ringing license from the Icelandic Institute of Natural History, Reykjavik (licensed to A. Petersen, the responsible person in Iceland) and no special permit was required. No birds were sacrificed, and no birds died or suffered (wounds etc.) during the study.

### Chemical analyses

Analyses of plasma samples were performed at the Great Lakes Institute for Environmental Research (GLIER), University of Windsor, Canada. Leat *et al*. [11] and references therein provides all details regarding the chemical analyses. In this analysis we used wet weight concentrations of 39 OC compounds; 29 polychlorinated biphenyl (PCBs) congeners and 10 OC pesticides: dichlorodiphenyltrichloroethane (DDT), dichlorodiphenyldichloroethylene (*p*,*p’-*DDE), dichlorodiphenyldichloroethane (*p*,*p’-*DDD), hexachlorobenzene (HCB), oxychlordane, mirex, β-hexachlorocyclohexane (β-HCH), *trans*-nonachlor, *cis*-nonachlor, *cis*-chlordane. In addition, three polybrominated diphenyl ether (BDE) congeners were used in the analysis: BDE-47, -100 and -153.

### Statistical analyses

Statistical analyses were carried out using general linear models and generalized linear models (Logistic regression) in the GENMOD procedure (Type 1 and 3 statistics) of SAS [24]. There were strong correlations (0.72< *R* ^2^ < 0.92) between the compounds that made up the bulk of the OC loads; *i*.*e*. PCBs, mirex, DDE, HCB and oxychlordane, comprising ~93–98% of the OCs in different colonies. Similarly, the BDE congeners were strongly correlated (*R* ^2^ > 0.46). Moreover, we also ran a principal component analysis on both OCs and BDEs, but PC1 scores were highly correlated to ∑OC and ∑BDE (0.81 < *R* ^2^ < 0.99). Hence, to avoid excessive statistical testing we chose to use ∑OC and ∑BDE in the analyses, which enabled us to relate effect relationships with contaminants directly to variations in concentrations. ∑OC and ∑BDE values were log_e_ transformed to approximate a normal distribution and achieve equal variance (assessed using Q-Q plots [25]). Relationships between fitness traits and POPs were analysed separately for each colony and year (including Bjørnøya 2009 and 2010), mainly because of the lack of overlap between the POP concentrations between the colonies (Table 2).

General linear models (GLM) were used to model traits with continuous dependent variables (hatching date, body condition of chicks at hatching, and chick growth-rate) whereas logistic regression was used to model traits with discrete variables (probability of egg loss and adult returning probability). Predictor variables were concentrations of ∑OC or ∑BDE and a set of covariates (body mass and body size [head and bill length]; blood lipid content (%); egg volume (for chick hatching condition); experimental treatment (feeding vs. controls), sex (for return-rate) and interactions. The effects of adult body condition and blood lipids on different traits were always included along with POPs. The independent variables were included in the final models when significant (*P* < 0.05). Following Rothman [26], Nakagawa [27], and Goldberg & Silbergeld [28], *P*-values were not adjusted (Bonferroni etc.) when testing the relationship between POPs and effect endpoints, but *P*-values from two-tailed tests are reported.

The data were checked for outliers and for hatching date one female hatching 10 days earlier than the next bird was removed for Bjørnøya in 2009. One A-chick was removed for Iceland and one for Bjørnøya 2010, whereas one B-chick was removed for Shetland and one for Bjørnøya 2010, because they were very heavy compared to their egg volume, suggesting that they had been fed before we were able to weigh them so we failed to record their hatching weights.

## Results

### Concentrations of POPs and breeding conditions

A total of 222 blood samples were collected; 161 females and 61 males. In 2009, concentrations of ∑OC were ~10 times higher in females at Bjørnøya than in Shetland, Iceland being 2.5 times higher than Shetland. For males the differences were somewhat smaller (Table 2). ∑BDE was also much higher at Bjørnøya in 2009 compared to the other colonies (Table 2). However, between 2009 and 2010 the mean ∑OC and ∑BDE concentrations at Bjørnøya declined by ~60% and ~80%, respectively (Table 2).

At Bjørnøya in 2009, the breeding conditions were very poor over the whole season (only 39% of the control nests hatched and only 17% [*n* = 5] of these chicks survived until 20 days of age), whereas in Iceland conditions degraded after hatching and were very poor during chick rearing (92% of control nests hatched, but only 5% of the chicks survived). On the contrary, breeding conditions in Shetland and at Bjørnøya in 2010 were good: ~90% of the control nests hatched, and 39% and 42% of the chicks survived, respectively (Tables 1 and 2).

### Reproductive effects of POPs

Compared with successful females, females losing their eggs at Bjørnøya in 2009 had higher plasma concentrations of ∑OC, with marginal significance (Mean ± SE: 2696±280 *vs*. 1984 ± 328 ng/g, wt. weight; Logistic regression: χ^2^_1, 39_ = 3.60, *P* = 0.058; estimate successful = -0.98± 0.54 SE), but not ∑BDE (*P* = 0.62).

**Fig 1:**
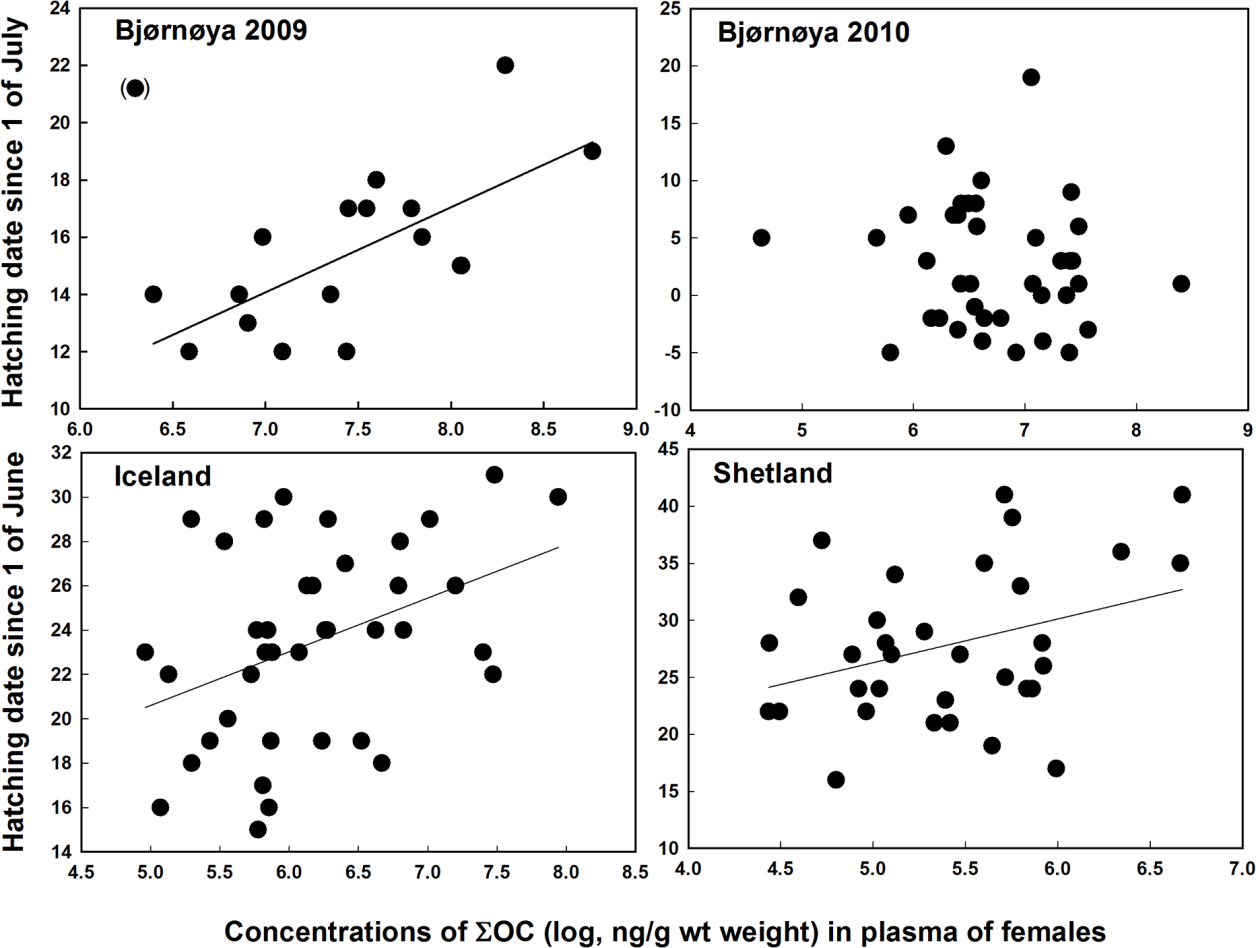
Relationships between female plasma concentrations of ∑OC (log) and hatching date in great skua colonies in the North-eastern Atlantic. *A*) Bjørnøya in 2009 (one individual in brackets has been excluded from the analysis), *B*) Bjørnøya in 2010, *C*) Iceland in 2009 and *D*) Shetland in 2009 (date since 1 of June [Shetland and Iceland] or 1 of July [Bjørnøya]).

**Fig 2:**
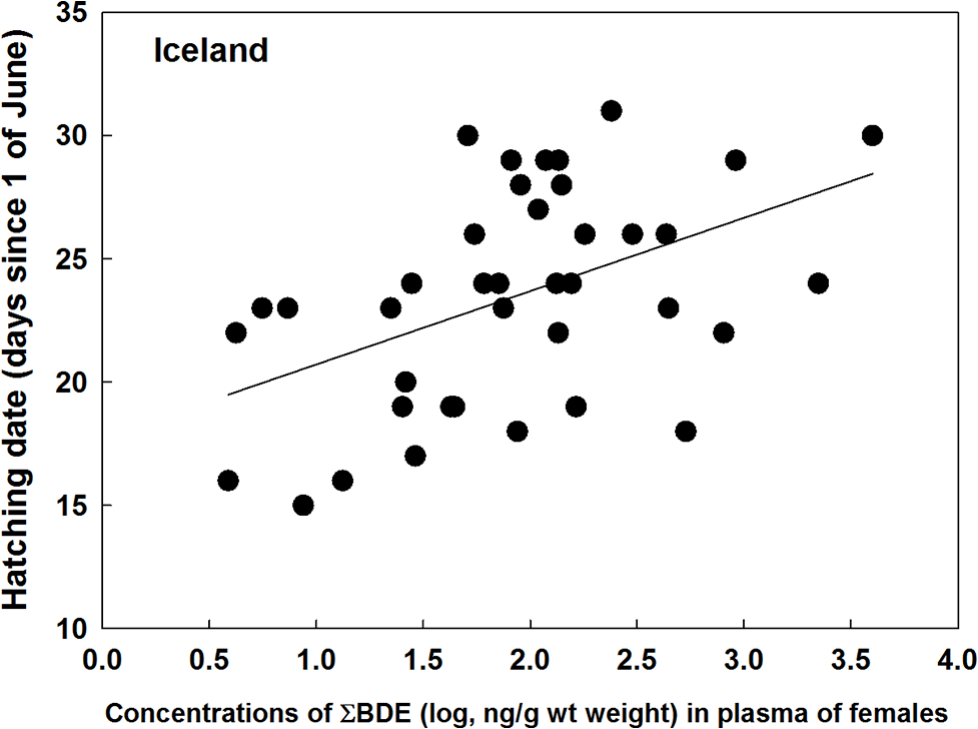
Relationships between hatching date (date since 1 of June) and female plasma concentrations of ∑BDE in great skuas breeding in Iceland in 2009.

The date of hatching varied greatly between the colonies, and also between years at Bjørnøya (Table 2). In Iceland there were significant positive associations between hatching date and concentrations of both ΣOC (*F*_1, 37_ = 6.91, *P* = 0.012, estimate = 2.42 ± 0.92 SE; Fig 1) and ΣBDE (*F*_1, 37_ = 10.87, *P* = 0.002, estimate = 2.97 ± 0.90 SE; Fig 2), whereas in Shetland a positive relationship was only found for ΣOC (*F*_1, 31_ = 4.08, *P* = 0.05, estimate = 3.83 ± 1.90 SE; Fig 1). At Bjørnøya in 2009, there was a strong positive relationship between ∑OC and hatching date (*F*_1, 15_ = 13.33, *P* = 0.002, estimate = 2.97 ± 0.81 SE), when removing one individual with very low concentration and a late hatching date (Fig 1). There was no significant relationship between POPs and hatching date at Bjørnøya in 2010 (*P* > 0.44; Fig 1).

**Fig 3:**
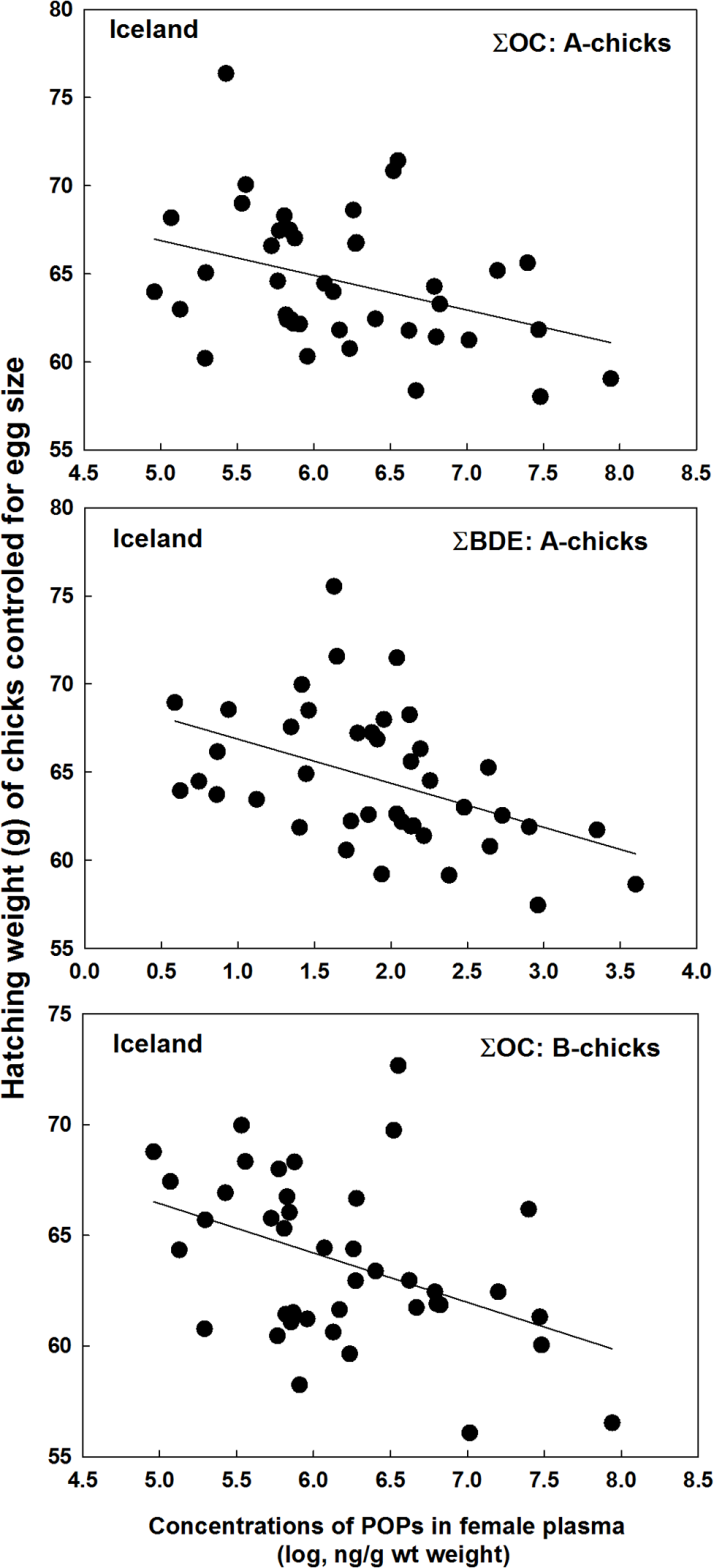
Hatching body conditions (hatching weight controlled for egg size) of the A-chicks and B-chicks in great skua broods in relation to the mothers’ plasma concentrations of ∑OC and ∑BDE. Data from Iceland in 2009.

**Fig 4:**
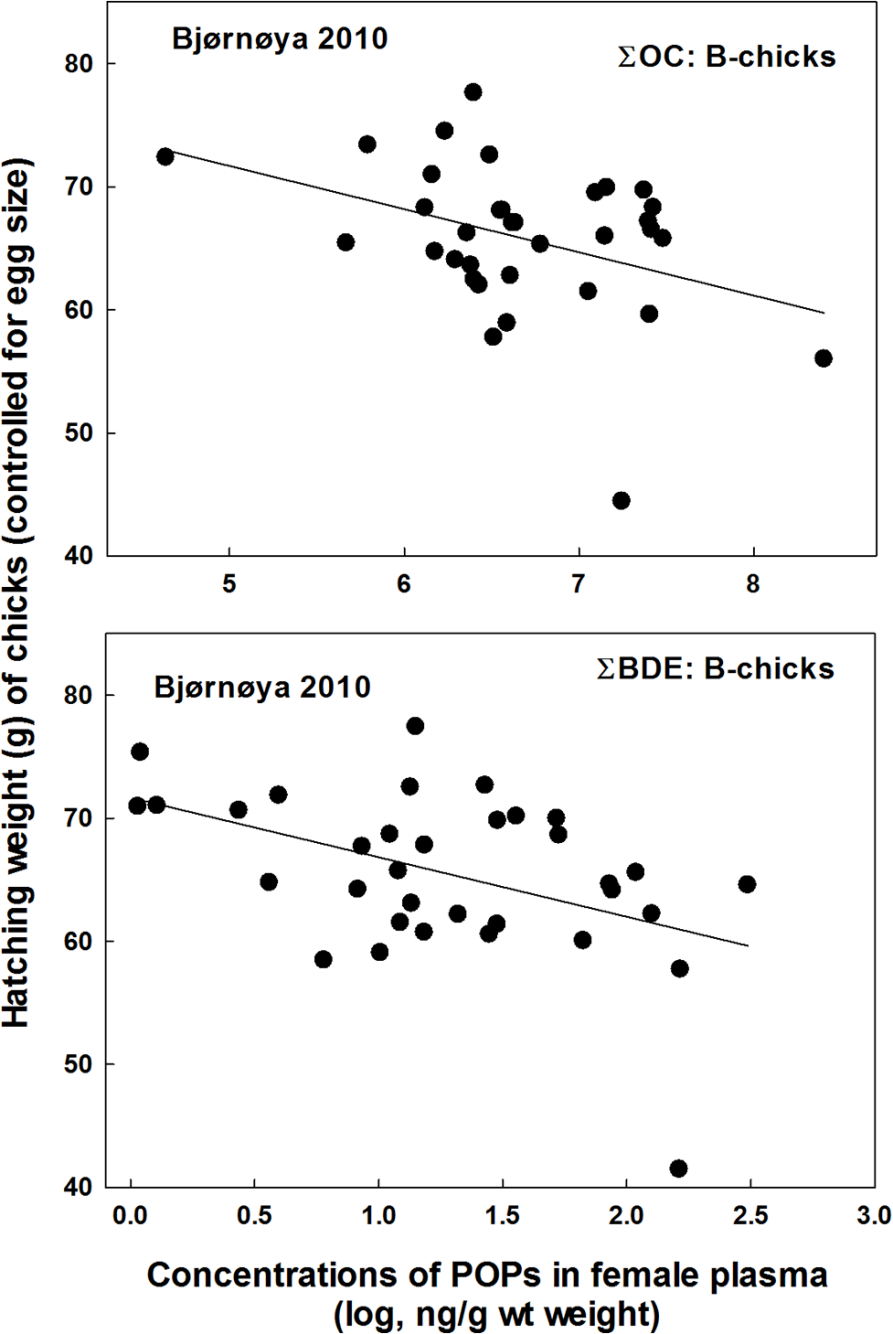
Hatching body conditions (hatching weight controlled for egg size) of B-chicks in great skua broods in relation to the mothers’ plasma concentrations of ∑OC and ∑BDE at Bjørnøya in 2010.

In Iceland, hatching weights of A-chicks were negatively related to the mothers’ concentrations of ∑OC (*F*_2, 34_ = 4.15, *P* = 0.045, estimate = -1.33 ± 0.65 SE) and ∑BDE (*F*_2, 34_ = 6.65, *P* = 0.014, estimate = -1.72 ± 0.67 SE; Fig 3), whereas for the B-chick they were only negatively related to ∑OC (*F*_2, 29_ = 4.37, *P* = 0.046, estimate = -1.41 ± 0.67 SE; Fig 3). At Shetland, hatching weights of chicks were not negatively related to any contaminants in the mothers (*P* > 0.50), whereas at Bjørnøya in 2010 the hatching weights of B-chicks were negatively related to both ∑OC (*F*_2, 26_ = 4.45, *P* = 0.045, estimate = -2.99 ± 1.42 SE) and ∑BDE (*F*_2, 26_ = 6.11, *P* = 0.02, estimate = -3.80 ± 1.54 SE; Fig 4). At Bjørnøya in 2009, however, there were no relationships between chick hatching weight and POPs (P > 0.43).

### Chick growth and supplementary feeding

All single chicks were A-chicks, but whether or not A-chicks had a sibling did not affect daily growth-rate until day 10 (0.16 < *P* < 0.56). In Shetland, there were no relationships between female POPs and chick growth (*P* > 0.50), but there was a positive relationship between daily growth-rate and female body condition for A-chicks (*F*_3, 17_ = 6.42, *P* = 0.02; estimate for controls = -5.17 ± 2.04 SE), and A-chicks receiving extra food grew faster than controls (*F*_*3*, 17_ = 6.34, *P* = 0.02, estimate = 0.042 ± 0.016 SE). For the B-chicks, however, there was no effect of female body condition (*P* = 0.13), but supplementary food increased their growth-rate (*F*_1, 6_ = 8.97, *P* = 0.02; estimate for controls = -5.25 ± 1.75 SE). At Bjørnøya in 2010 there was no association between contaminants and daily growth-rate of A-chicks (*P* > 0.25), but a positive effect of extra food (*F*_1, 32_ = 4.44, *P* = 0.042; estimate for controls = -3.11 ± 1.47 SE). For the B-chicks, however, there were no treatment effect but a significant interaction between treatment and both ΣOC (*P* = 0.015), showing a negative association between daily growth-rate and ∑OC in fed chicks (*F*_1, 7_ = 12.45, *P* = 0.001), but not in controls (*P* = 0.19). The interaction was not significant for ∑BDE (*P* = 0.09).

**Fig 5:**
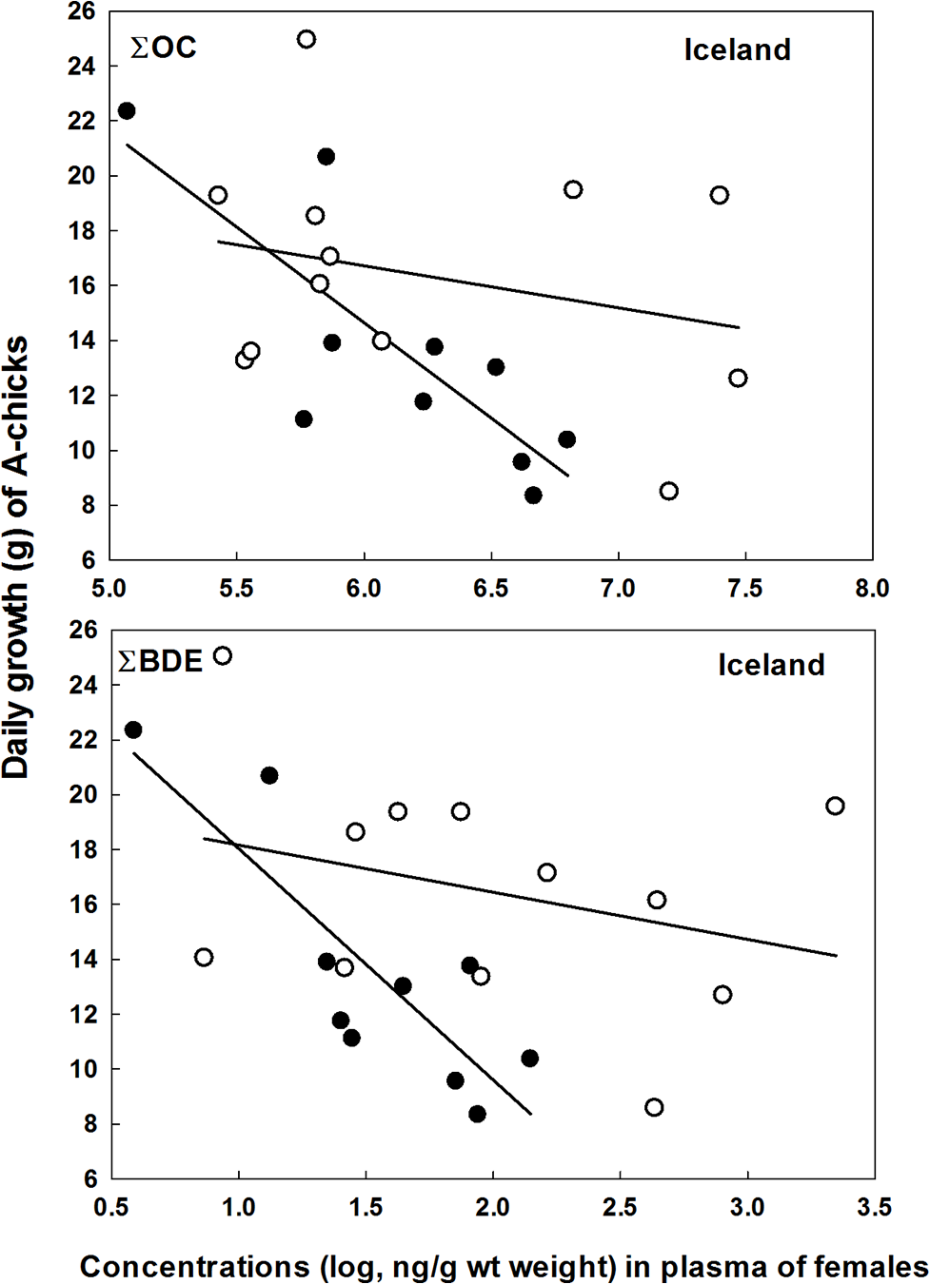
Daily growth rate (until age of 10 days) among A-chicks in great skua broods, receiving supplementary food (open circles) and controls (filled circles), in relation to the mothers’ plasma concentrations of ∑OC and ∑BDE. Data from Iceland in 2009.

**Fig 6:**
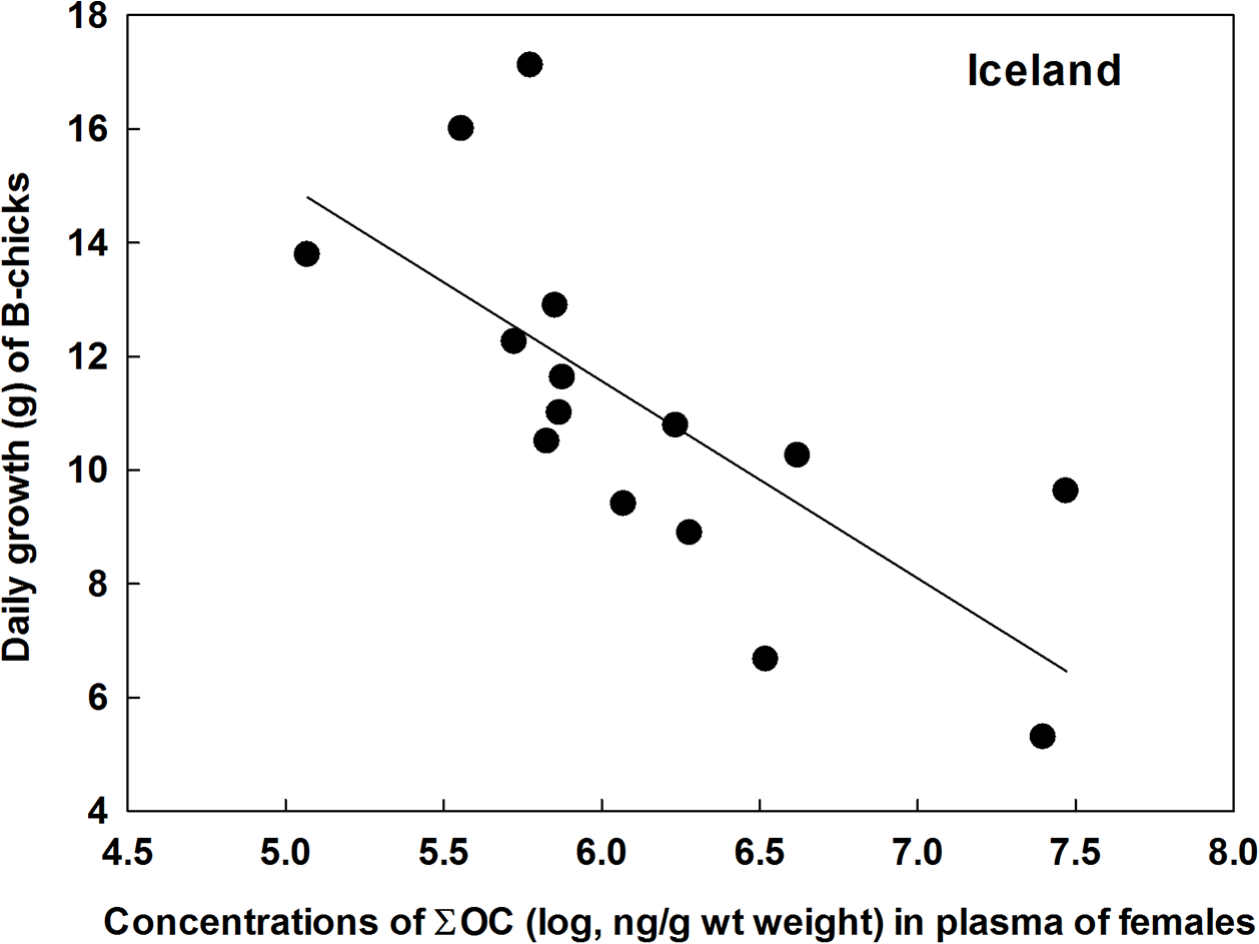
Daily growth rate (until age of 10 days) among B-chicks in great skua broods in relation to the mothers’ plasma concentrations of ∑OC. Data from Iceland in 2009.

In Iceland, there were significant interactions between treatment and both ∑OC and ∑BDE on daily growth of the A-chicks, indicating that extra food during the growth phase both increased growth rate and removed negative effects of contaminants (Table 3, Fig 5). For the B-chicks, however, there were no treatment effects (*P* > 0.26) and no treatment *x* POP interactions (*P* > 0.48), but a strong negative relationship between ∑OC and daily growth rate (*F*_1, 13_ = 14.2, P = 0.0025; estimate = -3.47 ± 0.93 SE, Fig 6). This was not significant for ∑BDE (*P* = 0.12).

**Table 3:** Relationships between daily growth rate of A-chicks (until day 10 of age) and persistent organic pollutants (∑OC and ∑BDE) in the blood of female great skuas in nests receiving different treatment (supplied extra food and controls). Data from Iceland 2009.

**∑OC**			**DF**	**F**	**P**	**Estimates**	**SE**
	Intercept					23.99	8.62
	Treatment	Control	1	4.07	0.059	32.43	16.08
		Fed				0	
	∑OC		1	10.38	0.0047	-1.38	1.37
	Treatment * ∑OC	Control	1	4.64	0.045	-5.58	2.59
		Fed				0	
	Error		18				
**∑BDE**							
	Intercept					18.45	2.77
	Treatment	Control	1	2.79	0.11	7.98	4.78
		Fed				0	
	∑BDE		1	12.97	0.002	-1.54	1.3
	Treatment * ∑BDE	Control	1	6.19	0.023	-6.87	2.76
		Fed				0	
	Error		18				

### Adult return rate between years

**Fig 7:**
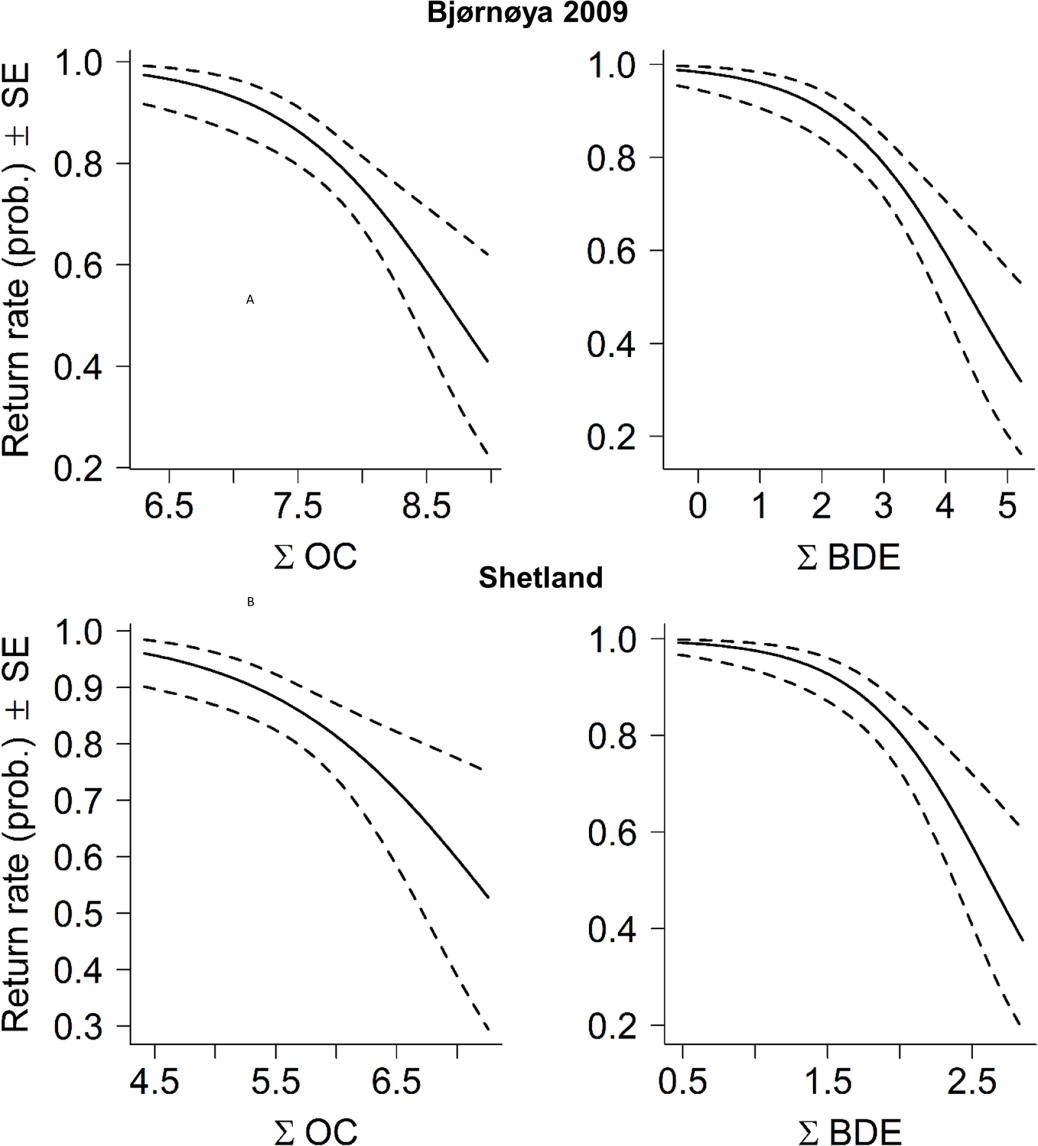
Returning probability (± SE) of great skuas between breeding seasons (A) at Bjørnøya (2009–2012) and (B) Shetland (2009–2010) in relation to plasma concentrations of ∑OC and ∑BDE (log, ng/g wt weight).

At Shetland, 86% (*n* = 43) of the birds breeding in 2009 returned in 2010; return was significantly and negatively related to ∑BDE (Logistic regression: χ^2^_1, 49_ = 5.72, *P* = 0.007; estimate = -2.27 ± 0.94 SE; Fig 7), and marginally significant for ∑OC (χ^2^_1, 49_ = 3.02, *P* = 0.07; estimate = -1.08 ± 0.62 SE; Fig 7). At Bjørnøya, 80% (*n* = 41) of the birds breeding in 2009 had returned by 2012. The return-rate was negatively related both to ∑OC (χ^2^_1, 49_ = 4.2, *P* = 0.02; estimate = -1.50 ± 0.73 SE; Fig 7) and ∑BDE (χ^2^_1, 49_ = 6.03, *P* = 0.005; estimate = -0.93 ± 0.38 SE; Fig 7). For Bjørnøya in 2010 (birds breeding in 2009 excluded) 88.5% (*n* = 46) had returned by 2012, and at Iceland 71% (*n* = 40) returned between 2009 and 2010; there were no relationships between return-rate and POPs for Iceland and Bjørnøya 2010 (*P* > 0.41). No significant relationships were found between return-rate and sex (*P* > 0.13) or body condition (P > 0.12), except at Iceland where females had lower return-rate than males (χ _1, 53_ = 4.98, *P* = 0.026; estimate = -2.24 ± 1.08 SE), and birds in good condition returned at a higher rate (χ^2^_1, 53_ = 8.49, *P* = 0.0036; estimate = 0.0113 ± 0.0044 SE).

## Discussion

In great skuas breeding in the North-Atlantic both observational and experimental data showed that contaminants were differently related to effects endpoints depending on population health and breeding conditions. The most pronounced fitness relationship, *i*.*e*. negative relationship with adult return-rate (a proxy for survival) was only found when population health was poor and reproductive investment high such as in Shetland, and in the healthy but heavily polluted Bjørnøya population following the extremely poor breeding season in 2009. Although the relationships between POPs and effect endpoints were correlational, this study indicated that the impacts of POPs were influenced by natural stressors; *i*.*e*. a multi-stressor situation.

### Variation in POP concentrations

POP concentrations differed greatly among the colonies, and in 2009 the Arctic colony at Bjørnøya, a known hotspot for POPs [29], was by far the most polluted. The reason for this is probably strong atmospheric and marine transport of POPs, and the high lipid dependence in Arctic wildlife resulting in high transfer of lipophilic POPs between trophic levels [30], [31]. However, there might also be dietary differences between the colonies. The reduction in concentrations at Bjørnøya between 2009 and 2010 is probably a result of improved feeding conditions and thereby lower metabolism of POP-containing body lipids [32]. Alternatively, skua diet may have changed, for example from seabirds to fish, the latter representing a lower trophic level than the former [33]. Moreover, in breeding seabirds males usually have higher plasma concentrations of POPs since females sequester some of their loads into the eggs [5], [18]. Equal POP concentrations in the sexes, such as skuas in Iceland, may indicate that females experience poor feeding conditions and deplete their lipid reserves during egg-laying and incubation; i.e. remobilizing more POPs [5].

### Breeding conditions and population health

Assessing food availability, and thus breeding condition, for an omnivorous predatory seabird that may switch between different diets is extremely difficult. However, reproductive success and food availability are closely linked in seabirds [34, 35]. Thus, whether nests successfully hatch and chicks survive are good proxies for food availability in long-lived seabirds; *i*.*e*. it affects both the parental motivation and ability to complete a reproductive attempt, and there is a trade-off between the chances of successful fledging of offspring and the costs to own survival [36]. Based on hatching success and early chick survival the differences between colonies were large. At Bjørnøya in 2009 conditions were very poor and >60% of the nests failed, and hardly any chicks reached 20 days of age, whereas in Iceland conditions seemed good during incubation (only 7% of eggs /nests lost), but only 5% of the chicks survived to 20 days of age. In Shetland, in 2009 there was an unusual situation compared to previous years since breeding conditions were very good [13] [14], [15], and the skuas subsequently invested heavily in reproduction, and gained relatively high reproductive output. This investment probably put high strain on the birds, reducing their body condition compared to the other colonies (body mass controlled for size; *P* = 0.02). The conditions were also good at Bjørnøya in 2010 (see Tables 1, 2).

Three biomarkers of health (the stress hormone corticosterone assessed in feathers, plasma oxidative stress and immunoglobulin levels) showed that the Shetland population was in relatively poor physiological health compared to the other colonies (Table 1) [16], and body condition of both males and females was also lower than in the other colonies (Table 2). The poorer health at Shetland probably depends on birds suffering sustained nutrient stress for many years indicated by consistently low breeding success, decreasing breeding numbers and survival rates, in addition to increasing rates of nonbreeding [37], [38]. In contrast, the Bjørnøya population has increased from a few birds in the 1970s to ~350 pairs at present (H.Strøm, unpublished data), and thus the population probably consists of younger individuals than in Shetland. Unfortunately, little is known about the population development in Iceland, but the biomarkers suggested that the population is in relatively good physiological condition, although slightly poorer than at Bjørnøya [16].

### Ecological effect endpoints

The reproductive parameters examined here (hatching date, hatching success and the body condition of the chicks at hatching) are closely linked to individual fitness. For example late hatching birds often suffer lower reproductive output [39], [40], whereas chicks in poor condition are less likely to survive [41], [42], [43]. There were positive associations between hatching date and female POPs in all colonies, the estimated difference between high and low polluted birds being about one week, except at Bjørnøya in 2010 (Fig 2). However, interpretation of these relationships should be cautious, since reproductive timing may be influenced by a number of factors (food availability and quality, body condition, migration timing etc.), which could confound the relationships with POPs. If POPs are causing the relationships the impact may be through actions on both the female and/or the developing embryo. Effects of POPs on the female may be through specific mechanisms, for example by disrupting reproductive hormones [44], [45], [46], or by increasing general stress [19], [47]. It might also be that chicks suffering high maternal transfer of POPs develop more slowly. However, this is not the only possible explanation since later hatching with increasing POPs was also found in Shetland at low POP concentrations, but not in Bjørnøya in 2010 with relatively high concentrations. Delayed hatching with increasing concentrations of POPs in female blood has also been found in south polar skua (*Catharacta maccormicki*) breeding in Antarctica [18] and in great black-backed gulls in Norway [5].

Some POPs may increase metabolism in developing embryos and reduce the body condition of the chick at hatching [17], [48], and such effects must result from maternally transferred contaminants. Transfer of lipophilic POPs tends to be proportional to the mother’s load, which has also been found in great skuas [23]. Reduced chick body condition was found in Iceland and Bjørnøya in 2010 at intermediate POP levels. In Shetland, however, the levels may have been too low to cause measurable impacts. At Bjørnøya in 2009 there was no significant relationship between POPs and chick body condition, although levels of POPs were high. However, breeding conditions were so poor that less than 40% of the clutches hatched, probably only from high quality birds and sample size was much lower than in the other colonies. Moreover, females with hatching eggs at Bjørnøya in 2009 had lower POP loads than failing ones suggesting either embryo toxicity or effects directly on the females.

Further evidence for state dependent impacts of POPs on great skua reproduction was the effects of supplementary feeding. Observational evidence that POPs are having different impacts on chick growth depending on energetic stress has been found in the glaucous gull [9], but this is the first feeding experiment to test such relationship in seabirds. In the nutrient-stressed Icelandic population there was a strong negative relationship between female POP loads and daily growth-rate up to day 10 in the A-chicks. Supplementary food increased the daily growth rate of chicks in all colonies, but in Iceland the treatment increased the survival time of chicks and removed negative relationships with POPs (Fig 5). The mitigation effects may operate on the females, *i*.*e*. extra food makes them better able to cope with POP stress, and thus increasing reproductive effort. However, in the chicks receiving extra food, the stress caused by maternally transferred POPs may be reduced, and they grow better as a result. That is, if food is scarce, high loads of POPs are transferred to the eggs since contaminated body lipids may be used for egg-production, reducing the chick condition at hatching [17], [48]. Little food during growth may then reinforce the negative impact of POPs. The lack of POP effects on A-chicks in Shetland and at Bjørnøya in 2010 was probably a result of good natural feeding conditions. For the B-chicks in Iceland there was a strong negative relationship between the mother’s ∑OC concentrations and daily growth-rate, but no effect of supplementary feeding. The reason for this is probably that the parents were favouring the A-chicks during strained feeding conditions, a brood-reduction strategy commonly found in various bird species [49]. Alternatively B-chicks may have lower viability and competitiveness than A-chicks. One clearly unpredicted result was the negative association between POPs and daily growth-rate in fed, but not control, chicks at Bjørnøya in 2010. We have no explanation for this, but it could be caused by random effects and small samples; notably one low and one high growth/POP individual caused the significant result.

It has previously been demonstrated that pollution loads measured in blood were associated with reduced return-rate (survival) between seasons in glaucous gulls at Bjørnøya [8], in great black-backed gulls [5], and lesser black-backed gulls (*Larus fuscus*) [50] in Norway. In Shetland where birds in poor health invested heavily in reproduction, and at Bjørnøya following the poor 2009 breeding season, birds with high POP levels were less likely to return. Return-rate between years has been used as a proxy for survival [8], but it will also be influenced by the probability of non-breeding. We do not know whether birds died or stayed in other areas, but site-fidelity of skuas to their nesting territories is very high [51], and the territories were thoroughly checked for returning birds. For Bjørnøya in 2009, we also added two extra years searching for returning adults, so we are confident that the birds not returning between 2009 and 2010 had died or at least had permanently abandoned their colony. This implies that the most severe effects of POPs (reduced survival) in skuas may occur both at high pollutant stress levels as found on Bjørnøya in 2009, but also in weakened individuals in the population which invested heavily in reproduction [52] even if POP concentrations are low. At intermediate pollution loads, however, there was no association between POPs and return-rate, but there was a body condition effect on Icelandic females, suggesting that body condition may also play a role. An important aspect of this is, however, that none of the other variables tested for, namely body condition or blood lipid content, could explain the relationships with POPs found in this study.

### Effects of OCs or BDEs

Concentrations of POPs in plasma are readily available to sensitive tissues, such as the brain, liver and kidneys and thus a good metric to assess potential effects of POPs. OCs and BDEs are lipophilic and thus relatively highly correlated, also in this study (*R* ^2^ ~ 0.20). It is thus difficult to distinguish between these compound groups when effects are concerned [53]. We note, however, the great difference in concentrations; i.e. mean ∑OC were ~50–240 times higher than ∑BDE in the different colonies (Table 1). A natural prediction is thus that ∑OC has the highest effect potential. However, for the 11 significant effect endpoints, 6 were most strongly associated to ∑BDE and 5 to ∑OC. We also noted the consistency between Shetland and Bjørnøya in 2009, where return-rate showed a stronger relationship to ∑BDE than ∑OC. The literature on toxicity of OCs and BDEs in birds seems to give little support for the assumption that BDEs are much more toxic than many OCs [19], [54]. At present it is thus not possible to conclude on this matter.

Due to the difficulties of manipulating POPs in wildlife, most ecotoxicological studies are correlational with regard to POP effects, and our study is no exception. It is, however, to our knowledge the first empirical study (based on both observation and experiment) that examines the potential ecological impact of lipophilic POPs in the same top predator seabird species on a large geographical scale. The findings indicate that the adverse effects of POPs are state dependent, varying both with concentrations, the breeding conditions that the birds are experiencing, with population health, and probably also with reproductive investments by the birds. Hence, seabirds such as the great skua may be suffering from multiple stressors with regard to pollution effects. The implications are that although the environmental concentrations of many POPs are decreasing, the impact of toxicants may not decrease at the same pace. This is worrying because the marine environment is under increasing pressure from exploitation and climate change, and many seabird species are declining worldwide. This should raise concern because although many of the POPs are decreasing, new compounds are still entering ecosystems at an increasing rate. Thus cocktail effects of different environmental toxicants may be severe in seabirds, and be a potential additional stress to the breeding populations.
